# Supplementary material for: Role of Annexin A1 Secreted by Neutrophils in Melanoma Metastasis
Source: Cells. 2023 Jan 27;12(3):425. doi: 10.3390/cells12030425 (PMC9913423; doi:10.3390/cells12030425)
Supplement: Supplementary file 1 [file cells-12-00425-s001.zip › cells-2130978-supplementary.pdf]

Supplementary Materials

Methods

1. Cell Viability Assay by -(4,5-dimethylthiazol-2-yl)-2,5-Diphenyltetrazolium Bromide

Melanoma cells were seeded at 2.5 × 10^4 cells/well in 24-well plates and incubated in the absence or presence of different concentrations of the cyclosporine H or WRW_4 and cultured for 24 h. Following the incubation period, the medium was carefully removed, and 300 μL of 3-(4,5-dimethylthiazol-2-yl)-2,5-diphenyltetrazolium bromide (MTT, 0.5 mg/mL; #M5655, Sigma-Aldrich) was added into each well. Cells were maintained at 37 °C for 3 h, after which the supernatant was removed and 200 μL of dimethyl sulfoxide (DMSO; #276855, Sigma-Aldrich) was added into each well, and the mixture was homogenized for 15 min. Absorbance was determined using a spectrophotometer at 575 nm (SpectraMax M Series, Molecular Devices, San Jose, CA, USA). The results were expressed as the percentage of viable cells relative to NT cells (control).

2. Obtention of Melanoma-Conditioned Medium—MCM

To obtain the melanoma-conditioned medium (MCM), B16F10 cells were plated and cultured to obtain up to 80% of confluence, after which, the medium was changed and melanoma cells were cultured for 48 hours. The medium was recovered, filtered through a 45 μm filter (Corning), stored at -80 °C, and used according to assays.

3. Determination of Chemical Mediators

The levels of chemical mediators were quantified in neutrophil supernatant and bronchoalveolar lavage fluid (BALF) by ELISA using commercial kits according to the manufacturer's instructions. We used mouse CXCL1 KC (#555252 BD Biosciences, Franklin Lakes, NJ, USA), mouse MCP1 (#555260, BD Biosciences), mouse Arginase-1 (#MBS935646, MyBiosource), mouse VEGF-A (#SMV00 R&D systems, Minneapolis, MN, USA), mouse IL-10 (#555252, BD Biosciences), mouse TGF-β (#BMS608/4 e-Bioscience, San Diego, CA, USA), and mouse MMP-9 (#DY6718 R&D systems).

4. Bronchoalveolar Lavage Fluid (BALF)

To recover BALF, mice were submitted to tracheal cannulation, and the lungs were flushed twice with phosphate-buffered saline (PBS, 1.5 ml total volume) [1]. The collected BALF was centrifuged (1500 rpm for 15 min at 20 °C), and the resulting cell-free supernatant was recovered for determination of TGF-β and MMP-9 by ELISA using commercial kits (TGF-β (#BMS608/4 e-Bioscience, San Diego, CA, USA) and MMP-9 (#DY6718 R&D systems). The levels of mediators were normalized against protein concentration determined by Bradford (#5000204 Bio Rad, Hercules, California, USA).

5. Western blotting for AnxA1 cleavage detection

Neutrophil supernatant was resolved in 15 % SDS–polyacrylamide gel and blotted onto the PVDF membrane. The membrane was blocked with 5 % milk powder in TBS-0.5%Tween 20 for at least 1 hour at room temperature. For AnxA1 detection, the membrane was incubated with AnxA1 (1:1000; #71-34000 Invitrogen). After this, the membrane was washed and incubated for 1 hour with horseradish peroxidase (HRP)-conjugated secondary antibody at room temperature. Finally, blots were developed using the ECL system (GE-Healthcare, Chicago, Illinois, USA).

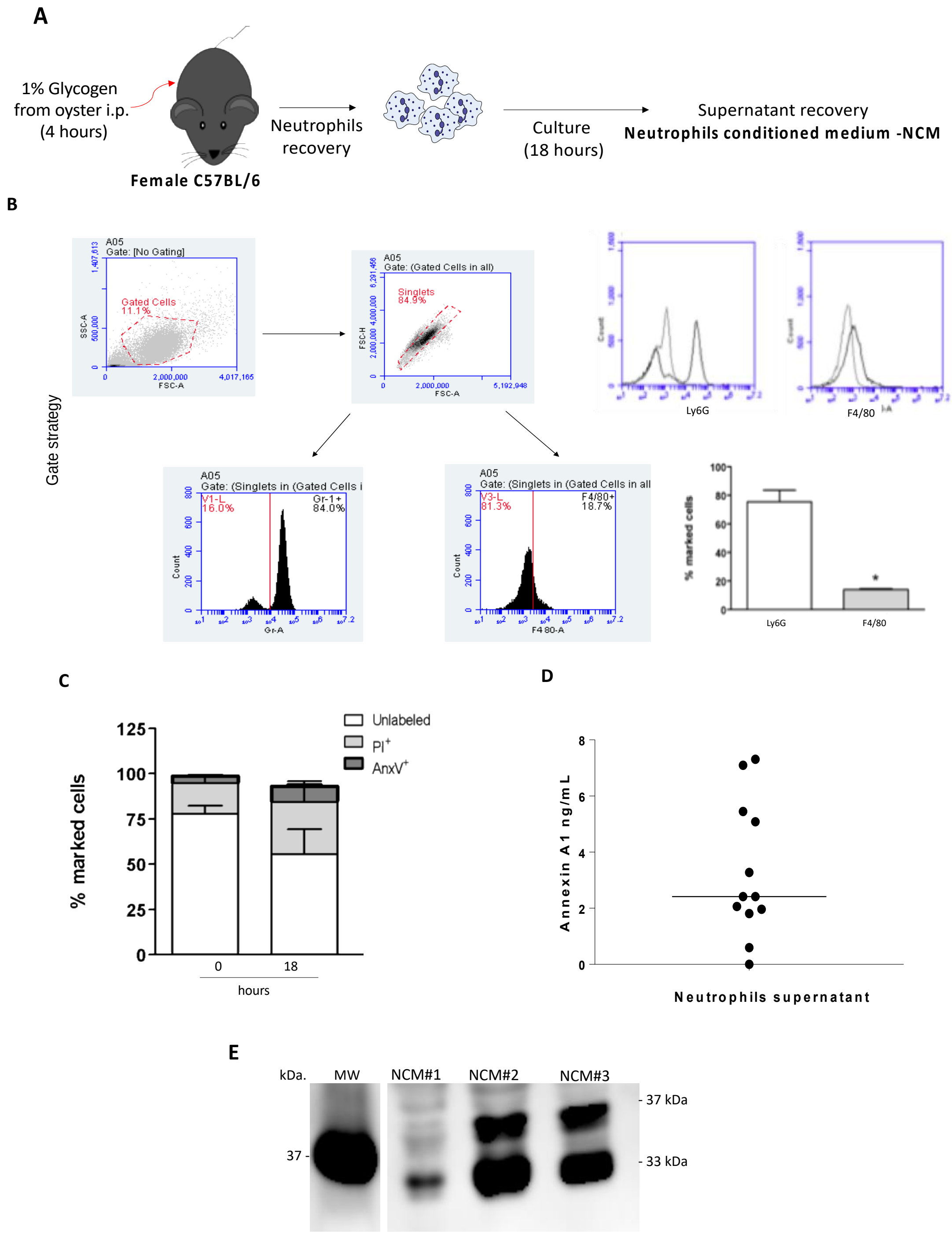

Figure S1. Obtention and characterization of mouse peritoneal cells. (**A**) Experimental design. (**B**) Labeling for Ly6G and F4/80 markers for neutrophils and macrophages, respectively. (**C**) Cell viability assessed by propidium iodide (PI) and Annexin V (AnxV). The data represent the average + SEM of at least five independent experiments. (**D**) Levels of AnxAl secreted by neutrophils (1×10^6) after 18 h of culture (n = ll). (**E**) Cleavage of AnxAl in the neutrophil supernatant detected by Western blotting (n = 3 samples).

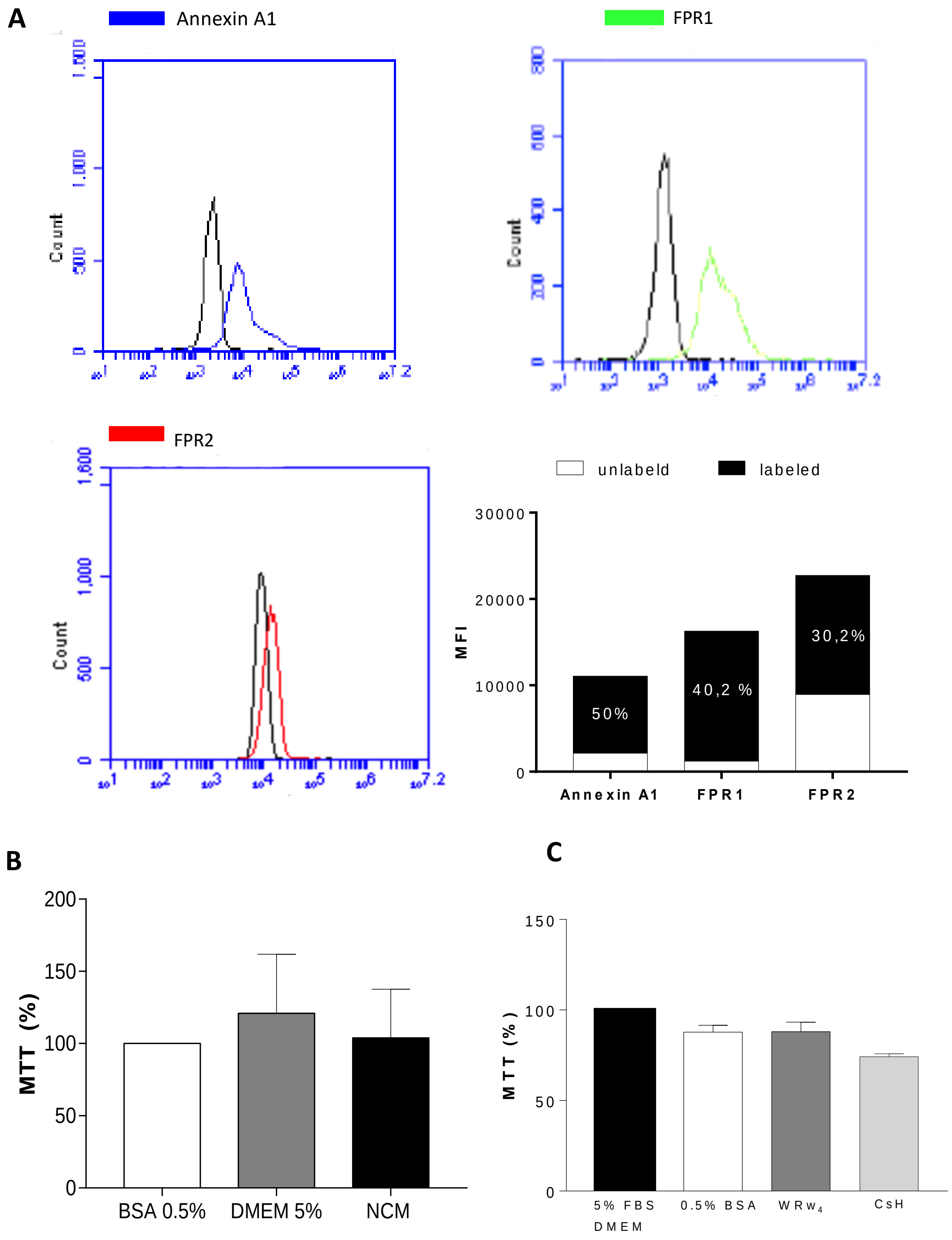

Figure S2. Characterization of melanoma cells. (**A**) AnxAl and FPR receptors expression in melanoma cells assessed by flow cytometry. (**B**) Cell viability in the presence of DMEM 5% or NCM incubated for 24 hours determined by MTI'. (**C**) Evaluation of cell viability in the presence of the FPR inhibitors maintained in DMEM supplemented with 0.5 % BSA for 24 hours. The data represent the average ±SEM of at least 5 five independent experiments.

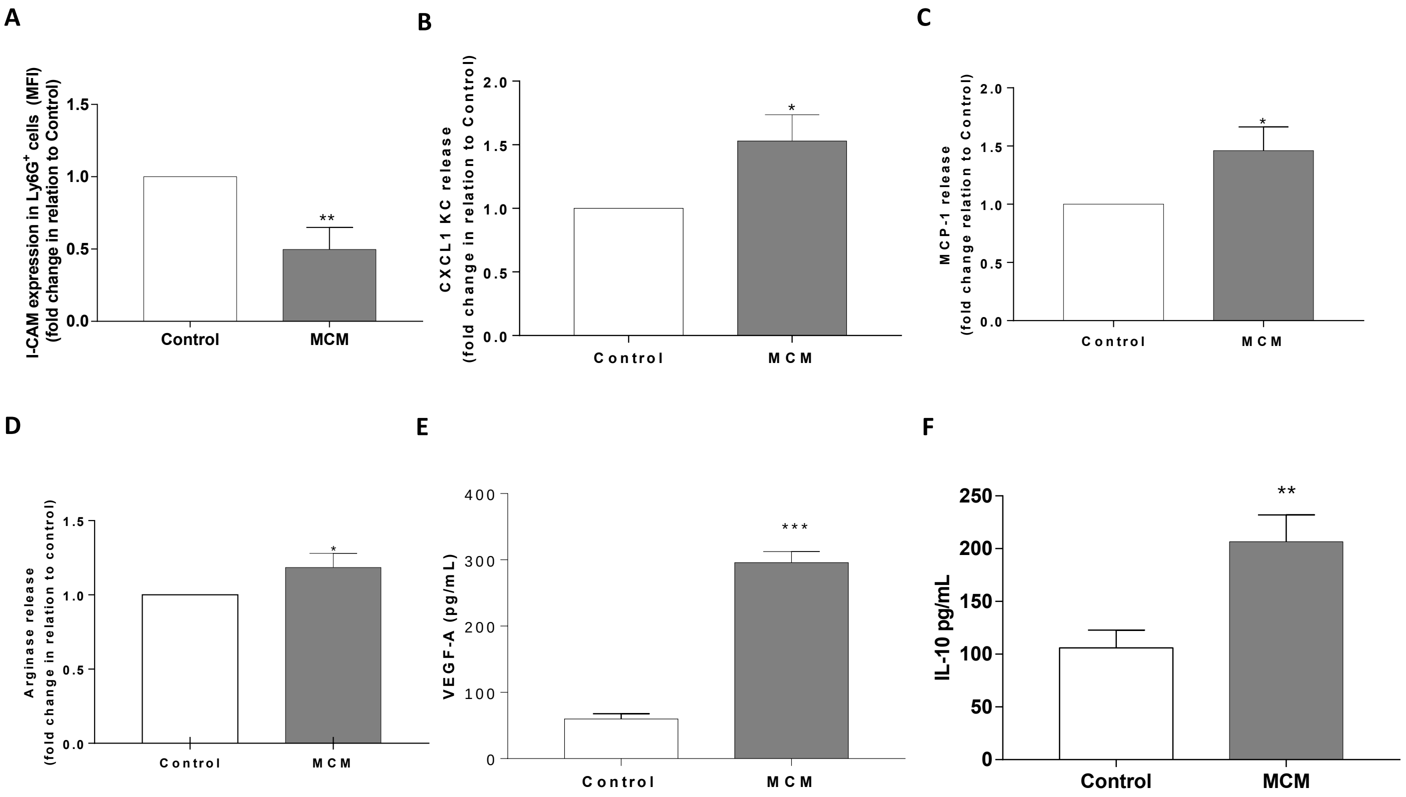

Figure S3. Neutrophil profile after 24 hours of culture with melanoma-conditioned medium (MCM). (**A**) I-CAM expression in neutrophils (Ly6G+). (**B**) CXCLl KC, (**C**) MCPl, (**D**) Arginase, (**E**) VEFGF, and (**F**) IL-10 secretion by neutrophils cultured in the absence (control) and presence of MCM for 24 hours. The data represented the average ± SEM of at least 5 five independent experiments. * $p < 0.05$; ** $p < 0.001$ vs. control.

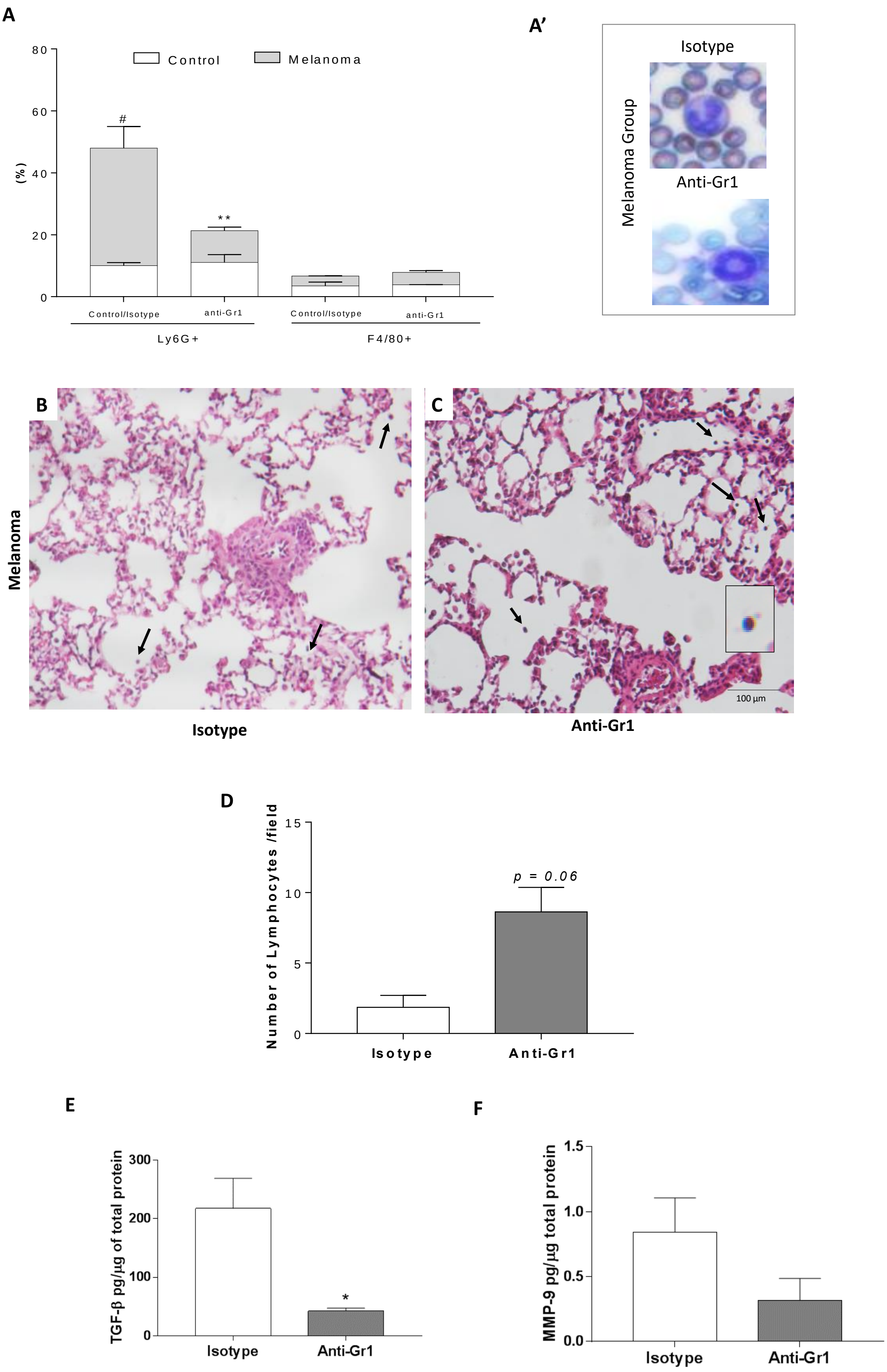

Figure S4. (**A**) Evaluation of the circulating neutrophils (Ly6G+) and monocytes (F4/80) on the 21st day after melanoma cells injection. # p <0.05 vs. control; ** p <0.01 vs. melanoma. (**A'**) Morphology of circulating neutrophils stained by Giemsa. (**B,C**) Morphological analysis by H&E staining showing an increase of lymphocytes in lung melanoma metastasis from isotype or anti-Gr1-treated mice . Insert: lymphocyte. (**D**) Number of lymphocytes counted in lung melanoma metastasis (5 fields counted *per* section). (**E,F**) Amounts of TGF-β and MMP-9 found in the BALF from isotype or anti-

Gr1-treated mice. The data represent the average + SEM of at least 3-5 five animals per group. * $p < 0.05$ vs. isotype.

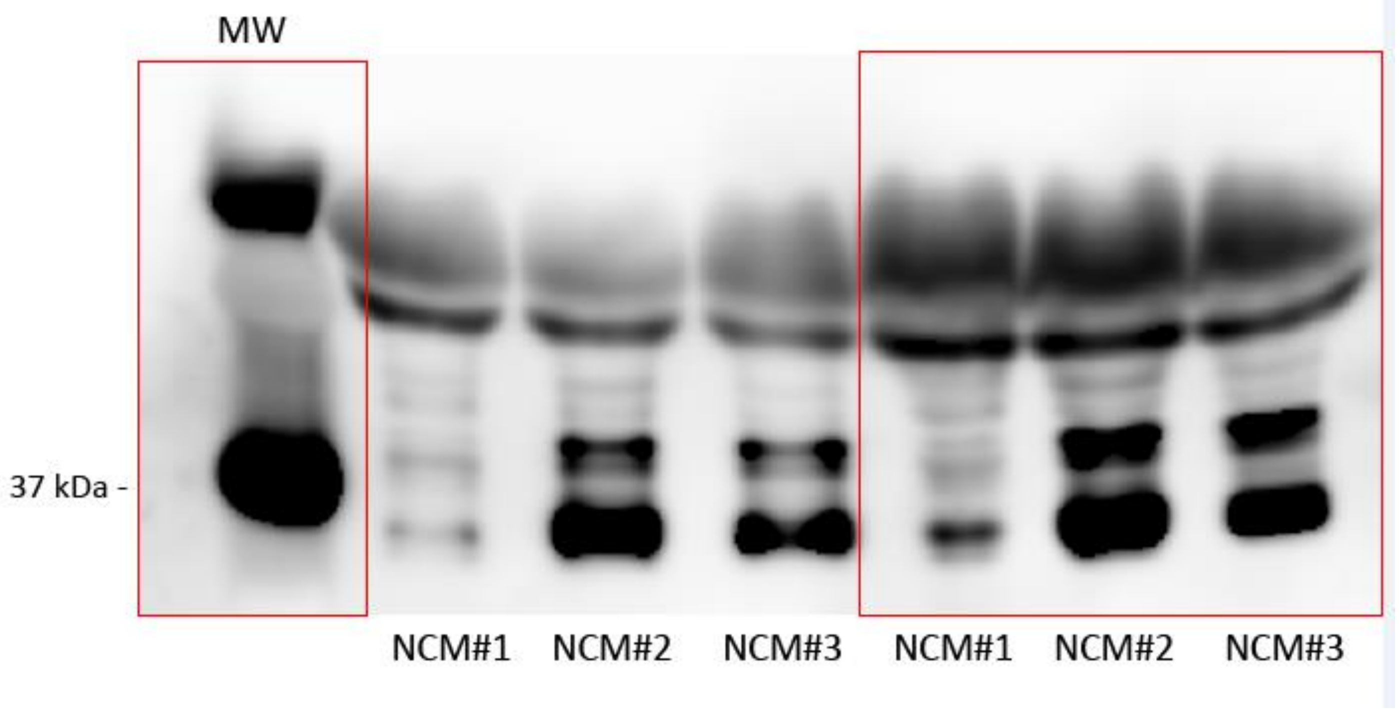

Figure S5. Uncropped gel - Western blot assay was performed to evaluate whether the AnxAl detected by ELISA was cleaved. Red square—image used in Figure S2E. The uncropped blot is related to neutrophil-conditioned medium (NCM #1; NCM #2; NCM#3) obtained from neutrophils collected from 3 animals and cultured. The gel presents samples at different total protein concentrations (left: 20 µg; right: 40 µg).

References

1. Rodrigues da Silva, M.; Schapochnik, A.; Peres Leal, M.; Esteves, J.; Bichels Hebeda, C.; Sandri, S.; Pavani, C.; Ratto Tempestini Horliana, A.C.; Farsky, S. H. P.; Lino-Dos-Santos-Franco, A. Beneficial effects of ascorbic acid to treat lung fibrosis induced by paraquat. *PLoS ONE* **2018**, *13*, e0205535.
